# Antimicrobial Susceptibility Patterns of Uropathogens: A Retrospective Study at a Primary Care Hospital in Ghana

**DOI:** 10.1002/hsr2.71029

**Published:** 2025-07-09

**Authors:** Samuel Kyeremeh Adjei, Prosper Adjei

**Affiliations:** ^1^ Department of Internal Medicine Methodist Hospital Wenchi Bono Region Ghana; ^2^ School of Public Health and Allied Sciences Catholic University of Ghana Fiapre‐Sunyani Bono Region Ghana

**Keywords:** antibiotic susceptibility, antimicrobial resistance, Ghana, urinary tract infections, uropathogens

## Abstract

**Background and Aims:**

Urinary tract infections are among the most common infections globally, with increasing rates of antibiotic resistance complicating their management. This study aimed to determine the prevalence, bacteriological profile, and antibiotic susceptibility patterns of uropathogens isolated from urine samples at Methodist Hospital, Wenchi, Ghana.

**Methods:**

A retrospective study was conducted using data on urine culture and antibiotic susceptibility testing obtained from the Microbiology Unit of the Laboratory Department between March 2024 and January 2025.

**Results:**

A total of 504 urine samples were analyzed, revealing a prevalence of urinary tract infections at 45.2%. The predominant pathogens were Gram‐negative bacteria, with *Klebsiella spp* (12.3%) and *Escherichia coli* (10.1%) being the most frequently isolated organisms. *Candida spp* were also identified in 5.8% of the samples. Females accounted for 74.6% of the infections, with the highest prevalence observed among individuals aged 20–40 years. Antimicrobial susceptibility testing demonstrated significant resistance among bacterial isolates, with *Klebsiella spp* showing the highest resistance to Beta‐lactams and Fluoroquinolones. Amikacin exhibited the lowest resistance rate (4.4%), making it a strong candidate for empirical therapy. Multi‐drug resistance (MDR) was demonstrated by 72.7% of bacterial isolates, with *Klebsiella spp* and *Escherichia coli* showing the highest MDR rates.

**Conclusion:**

The identified patterns of antimicrobial resistance highlight the critical importance of careful antibiotic selection to effectively manage urinary tract infections.

**Abbreviations**
AMK: amikacin
AMR: antimicrobial resistance
AU: augmentin
CEF: ceftriaxone
CFT: ceftazidime
CIP: ciprofloxacin
GEN: gentamicin
LEV: levofloxacin
MDR: multi‐drug resistance
NAL: nalidixic acid
NIT: nitrofurantoin
NOR: norfloxacin
PIP: piperacillin
TET: tetracycline
UTI: urinary tract infection

## 1 Introduction

Urinary tract infections (UTIs) represent one of the most prevalent types of infections acquired both in the community settings and hospitals, and they are increasingly associated with antibiotic resistance [1]. UTIs encompass a range of infections that occur in various parts of the urinary system, which include the kidneys, ureters, bladder, and urethra. The urinary tract is divided into two distinct sections: the upper tract comprising the kidneys and ureters, and the lower tract consisting of the bladder and urethra [2]. UTIs are caused by a variety of pathogens, including both Gram‐negative and Gram‐positive bacteria, in addition to specific fungal organisms [3]. Gram‐negative bacteria are predominantly observed, with *E. coli* accounting for the majority of bacterial uropathogens globally [4]. Other bacterial species implicated in UTIs include *Klebsiella pneumoniae*, *Proteus mirabilis*, *Enterococcus faecalis*, and *Staphylococcussaprophyticus* [5]. Annually, approximately 150 million individuals worldwide receive a diagnosis of UTI, leading to an estimated healthcare cost that surpasses 5 billion dollars [6].

The prevalence of the infection ranges from 40% to 50% in women, while approximately 5% of men are affected. This disparity can be linked to hormonal fluctuations associated with pregnancy and distinct anatomical characteristics, which render females more susceptible to UTIs [7, 8]. The prevalence may also increase with advancing age, presence of diabetes, catheterization, sexual activity, menopause, absence of circumcision, and issues related to the prostate [9]. Infections are also prevalent among young women who are sexually active, with an incidence rate surpassing 0.5 episodes per individual annually. Approximately 30% of this population experiences recurrent infections [10]. Data from sub‐Saharan Africa indicate diverse prevalence rates of UTIs across various populations and age demographics. Specifically, the prevalence is reported at 89.1% among female patients in Nigeria [1], 39.1% among adult outpatients in Uganda [11], 10.1% in Ghana [12], 26.7% among adult patients in Senegal [13], and 21.2% among children in Gambia [14].

The inappropriate use of antimicrobials in the treatment of UTIs plays a substantial role in the emergence of antimicrobial resistance (AMR). AMR is projected to result in approximately 700,000 fatalities annually worldwide [15]. Should the current trajectory persist, this figure could exceed 10 million deaths each year by the year 2050. In the African continent, without intervention, it is estimated that around 4.1 million individuals may succumb to treatment failures attributable to AMR by 2050 [16]. Recent data indicate that Western sub‐Saharan Africa faces the highest burden, with 27.3 deaths per 100,000 directly attributed to AMR and 114.8 deaths per 100,000 associated with AMR in 2019 [17]. Significant rates of AMR have been documented in West Africa, with Ghana exhibiting a prevalence of 80.1% [12], 60% in Senegal [13], and 9.0% in Gambia [18].

While global and regional trends in AMR are well‐documented, local surveillance studies are critical for understanding the distribution of uropathogens and their antibiotic sensitivity patterns in specific populations and healthcare settings. Studies examining the bacteriological profile of uropathogens not only provide valuable insights into the prevailing resistance mechanisms but also serve as a foundation for developing institutional antibiograms and guiding empirical therapy.

This single‐center retrospective study aimed to determine the bacteriological profile and antibiotic sensitivity patterns of uropathogens isolated from urine samples at a primary care hospital in Ghana.

## 2 Materials and Methods

### 2.1 Study Design and Setting

This retrospective study was conducted at the Microbiology Unit of the Laboratory Department of the Methodist Hospital, Wenchi, Ghana. Secondary data on urine culture and susceptibility testing from March 2024 to January 2025 were used for the study.

### 2.2 Study Population

The records of all patients with suspected UTI for whom urine culture and antimicrobial susceptibility tests were conducted during the specified period were included in the study. Patients whose records were missing essential information such as age, sex, bacterial isolate, and antibiotic susceptibility test results were excluded from the analysis.

### 2.3 Specimen Collection, Processing, and Identification of Isolates

Early morning midstream urine specimens were collected using leak proof sterile plastic containers. Aseptic techniques were strictly adhered to throughout the sample collection process. A calibrated inoculating loop with a volume of 0.001 mL was employed to inoculate each urine sample onto Cysteine Lactose Electrolytes Deficient (CLED) agar plates, which were subsequently incubated at 37°C for a duration of 18 to 24 h. Following the overnight incubation period, the colonies were enumerated to assess the bacterial load per milliliter of the urine sample [19]. A urine specimen was classified as positive if the cultured isolates exhibited a concentration of ≥10^5^ cfu/mL. Following a 24‐h incubation period, bacterial colonies demonstrating pure and significant growth were subsequently identified through examination of colonial morphology on media, chromogenic appearance, Gram staining and standard biochemical tests including, glucose and lactose fermentation, H_2_S production, catalase, oxidase, coagulase, urease, novobiocin, bacitracin, indole and citrate utilization tests [20]. All positive urine cultures that showed significant bacteriuria were identified using UTI DISCS from BIOMARK LABORATORIES (PUNE 411041, INDIA).

### 2.4 Antimicrobial Susceptibility Testing

Antimicrobial susceptibility testing was conducted using disc diffusion technique on Mueller Hinton II agar (Liofilchem) in accordance with the protocols established by the Clinical and Laboratory Standards Institute (CLSI) [21]. Antibiotics tested included Augmentin (30 mcg), Ciprofloxacin (5 mcg), Ceftriaxone (30 mcg), Gentamicin (19 mcg), Piperacillin (20 mcg), Nitrofurantoin (300 mcg), Nalidixic acid (30 mcg), Ceftazidime (20 mcg), Norfloxacin (20 mcg), Tetracycline (30 mcg), Amikacin (30 mcg) and Levofloxacin (5 mcg).

The diameter of the inhibition zone surrounding the disc was assessed using a digital metal caliper, and the isolates were categorized as sensitive, intermediate, or resistant in accordance with CLSI guidelines [21].

### 2.5 Data Retrieval, Processing and Analysis

Archived data on urine culture and susceptibility test results from the hospital's microbiology unit were gathered and then entered into Microsoft Excel 2021. Data elements were then checked for completeness and subsequently exported into IBM Statistical Package for Social Sciences version 25 (SPSS v25) for statistical analysis. The basic characteristics of the study population were illustrated using frequencies along with mean and standard deviation values.

### 2.6 Ethical Statement

Ethical approval as well as waiver of informed consent were obtained from the Research Ethics Committee of Methodist Hospital, Wenchi (Ethics approval reference MHW/RE/103). All data collected for the study were treated with utmost confidentiality.

## 3 Results

A total of 504 urine samples were subjected to laboratory analysis and examination for uropathogens during the study period. Out of this, 63.7% (*n* = 321) were samples collected from females. The mean age of participants was 40.2 (SD ± 20.8) and ranged from 1 to 107 years. Majority of the participants were in their twenties (22.4%) and thirties (20.2%) at the time of sample collection. The smallest proportions were observed among younger subjects and teenagers (Table 1).

**TABLE 1:** Socio‐demographics characteristics of study participants.

Characteristic	Frequency (*n*)	Percentage (%)
**Age (years)**		
Mean ± SD	40.2 ± 20.8	
Range	1–107	
**Age group**		
≤10	34	6.7
11–19	36	7.1
20–29	113	22.4
30–39	102	20.2
40–49	60	11.9
50–59	54	10.7
60–69	54	10.7
≥70	51	10.1
**Gender**		
Male	183	36.3
Female	321	63.7

Abbreviation: SD, standard deviation.

The overall prevalence of UTIs among the study population was 45.2% (228/504). Gram‐negative bacterium, *Klebsiella spp* was the most prevalent uropathogen identified, at a rate of 12.3% (*n* = 62). This was closely followed by *Escherichia coli* (10.1%, *n* = 51) and *Pseudomonas aeruginosa* (7.5%, *n* = 38) with gram‐positive bacteria, *Enterococcus spp* and *Staphylococcus epidermidis* being the least prevalent pathogens isolated. Interestingly, *Candida spp* accounted for 5.8% (*n* = 29) of pathogens isolated from urine samples (Table 2).

**TABLE 2:** Distribution of uropathogens isolated from urine samples of patients at Methodist Hospital, Wenchi, Ghana.

Name of Isolate	Frequency (n)	Percentage (%)
**Gram‐negatives**		
*Citrobacter spp*	15	3.0
*Enterobacter spp*	6	1.2
*E. coli*	51	10.1
*Klebsiella spp*	62	12.3
*P. aeruginosa*	38	7.5
*Proteus spp*	13	2.6
**Gram‐positives**		
*Enterococcus spp*	1	0.2
*S. saprophyticus*	5	1.0
*S. aureus*	5	1.0
*S. epidermidis*	1	0.2
*Streptococcus spp*	2	0.4
**Fungus**		
*Candida spp*	29	5.8

Abbreviation: *spp*, species.

A significant majority (74.6%, *n* = 170) of the total isolates originated from samples collected from female participants. Regarding specific pathogen or species distribution, the majority of isolates from *Klebsiella spp* (83.9%, *n* = 52) and *Escherichia coli* (72.5%, *n* = 37) were recovered from female samples. A comparable female predominance was noted among the different species of pathogens isolated (Table 3).

**TABLE 3:** Distribution of isolated pathogens stratified by Gender and Age groups of participants.

Name of isolate	Total	Gender		Age group							
		Male	Female	≤ 10	11–19	20–29	30–39	40–49	50–59	60–69	≥ 70
**Gram‐negatives**											
*Citrobacter spp*	15 (3.0)	4 (26.7)	11 (73.3)	0 (0.0)	1 (2.8)	2 (1.8)	5 (4.9)	1 (1.7)	2 (3.7)	1 (1.9)	3 (5.9)
*Enterobacter spp*	6 (1.2)	2 (33.3)	4 (66.7)	0 (0.0)	0 (0.0)	0 (0.0)	5 (4.9)	0 (0.0)	0 (0.0)	1 (1.9)	0 (0.0)
*E. coli*	51 (10.1)	14 (27.5)	37 (72.5)	0 (0.0)	2 (5.6)	20 (17.7)	7 (6.9)	3 (5.0)	8 (14.8)	8 (14.8)	3 (5.9)
*Klebsiella spp*	62 (12.3)	10 (16.1)	52 (83.9)	3 (8.8)	3 (8.3)	10 (8.8)	9 (8.8)	14 (23.3)	9 (16.7)	9 (16.7)	5 (9.8)
*P. aeruginosa*	38 (7.5)	14 (36.8)	24 (63.2)	1 (2.9)	3 (8.3)	5 (4.4)	1 (1.0)	5 (8.3)	3 (5.6)	8 (14.8)	12 (23.5)
*Proteus spp*	13 (2.6)	6 (46.2)	7 (53.8)	0 (0.0)	1 (2.8)	3 (2.7)	1 (1.0)	3 (5.0)	1 (1.9)	1 (1.9)	3 (5.9)
**Gram‐positives**											
*Enterococcus spp*	1 (0.2)	1(100)	0 (0.0)	0 (0.0)	1 (2.8)	0 (0.0)	0 (0.0)	0 (0.0)	0 (0.0)	0 (0.0)	0 (0.0)
*S. saprophyticus*	5 (1.0)	1 (20.0)	4 (80.0)	0 (0.0)	1 (2.8)	3 (2.7)	0 (0.0)	1 (1.7)	0 (0.0)	0 (0.0)	0 (0.0)
*S. aureus*	5 (1.0)	2 (40.0)	3 (60.0)	0 (0.0)	1 (2.8)	1 (0.9)	1 (1.0)	0 (0.0)	0 (0.0)	2 (3.7)	0 (0.0)
*S. epidermidis*	1 (0.2)	1 (100)	0 (0.0)	0 (0.0)	0 (0.0)	0 (0.0)	0 (0.0)	0 (0.0)	0 (0.0)	0 (0.0)	1 (2.0)
*Streptococcus spp*	2 (0.4)	0 (0.0)	2 (100)	0 (0.0)	0 (0.0)	1 (0.9)	1 (1,0)	0 (0.0)	0 (0.0)	0 (0.0)	0 (0.0)
**Fungus**											
*Candida spp*	29 (5.8)	3 (10.3)	26 (89.7)	0 (0.0)	3 (8.3)	8 (7.1)	6 (5.9)	1 (1.7)	5 (9.3)	2 (3.7)	4 (7.8)
**Total**	228 (45.2)	58 (25.4)	170 (74.6)	4 (1.8)	16 (7.0)	53 (23.2)	36 (15.8)	28 (12.3)	28 (12.3)	32 (14.0)	31 (13.6)

Abbreviation: *spp*, species.

Most of the uropathogens were cultured from urine samples collected from participants within the age range of 20–29 years (23.2%, *n* = 53). This was followed by participants aged 30–39 years (15.8%, *n* = 36) and 60–69 years (14.0%, *n* = 32). The lowest infection rate, recorded at 1.8% was observed in individuals aged 10 years or younger (Table 3).

The examination of the antimicrobial sensitivity pattern exhibited by the pathogens indicated that each bacterium was sensitive to at least two different antibiotics. Different bacteria exhibited distinct sensitivity patterns in response to various antibiotics. *Klebsiella spp* were mostly sensitive to Norfloxacin (42.2%) and least susceptible to Piperacillin (20.7%). For *Escherichia coli*, the highest susceptibility rate was 36.7% towards Piperacillin and least sensitive to Norfloxacin (Table 4).

**TABLE 4:** Proportion (%) of uropathogens demonstrating sensitivity (S) to antimicrobial agents.

Name of isolate	AU	CIP	CEF	GEN	PIP	NIT	NAL	CFT	NOR	TET	AMK	LEV
**Gram‐negatives**												
*Citrobacter spp*	0 (0.0)	3 (4.3)	3 (6.5)	5 (4.3)	1 (3.3)	8 (7.4)	2 (7.4)	0 (0.0)	2 (4.8)	3 (6.3)	14 (8.0)	14 (9.0)
*Enterobacter spp*	0 (0.0)	2 (2.9)	3 (6.5)	4 (3.5)	2 (6.7)	4 (3.7)	2 (7.4)	2 (7.7)	2 (4.8)	4 (8.3)	6 (3.4)	4 (2.6)
*E. coli*	7 (29.2)	17 (24.3)	15 (32.6)	34 (29.6)	11 (36.7)	32 (29.6)	7 (25.9)	7 (26.9)	9 (21.4)	13 (27.1)	43 (24.6)	41 (26.3)
*Klebsiella spp*	10 (37.8)	24 (32.1)	12 (22.4)	33 (28.7)	8 (20.7)	34 (30.3)	11 (34.0)	8 (23.8)	19 (42.2)	17 (32.0)	59 (33.3)	49 (30.8)
*P. aeruginosa*	3 (12.5)	13 (18.6)	9 (19.6)	24 (20.9)	7 (23.3)	19 (17.6)	3 (11.1)	8 (30.8)	8 (19.0)	9 (18.8)	31 (17.7)	28 (17.9)
*Proteus spp*	0 (0.0)	6 (8.6)	2 (4.3)	6 (5.2)	0 (0.0)	2 (1.9)	2 (7.4)	1 (3.8)	2 (4.8)	0 (0.0)	10 (5.7)	9 (5.8)
**Gram‐positives**												
*Enterococcus spp*	1 (4.2)	0 (0.0)	0 (0.0)	0 (0.0)	0 (0.0)	1 (0.9)	0 (0.0)	0 (0.0)	0 (0.0)	0 (0.0)	1 (0.6)	1 (0.6)
*S. saprophyticus*	1 (4.2)	3 (4.3)	1 (2.2)	4 (3.5)	1 (3.3)	4 (3.7)	1 (3.7)	0 (0.0)	1 (2.4)	1 (2.1)	5 (2.9)	5 (3.2)
*S. aureus*	2 (8.3)	3 (4.3)	2 (4.3)	3 (2.6)	1 (3.3)	3 (2.8)	1 (3.7)	1 (3.8)	1 (2.4)	2 (4.2)	5 (2.9)	4 (2.6)
*S. epidermidis*	0 (0.0)	1 (1.4)	0 (0.0)	1 (0.9)	0 (0.0)	1 (0.9)	0 (0.0)	0 (0.0)	0 (0.0)	0 (0.0)	1 (0.6)	1 (0.6)
*Streptococcus spp*	1 (4.2)	0 (0.0)	1 (2.2)	1 (0.9)	1 (3.3)	2 (1.9)	0 (0.0)	1 (3.8)	0 (0.0)	1 (2.1)	2 (1.1)	2 (1.3)
**Total**	25 (5.0)	72 (14.3)	48 (9.5)	115 (23.2)	32 (6.4)	110 (21.8)	29 (5.8)	28 (5.6)	44 (8.7)	50 (9.9)	177 (35.1)	158 (31.4)

Abbreviations: AMK, amikacin; AU, augmentin; CFT, ceftazidime; CIP, ciprofloxacin; CEF, ceftriaxone; GEN, gentamicin; LEV, levofloxacin; NIT, nitrofurantoin; NAL, nalidixic acid; NOR, norfloxacin; PIP, piperacillin; TET, tetracycline.

Regarding AMR, the highest resistance rates for *Klebsiella spp* were observed against Ceftriaxone (33.1%), Gentamicin (32.9%), Piperacillin (32.3%), and Levofloxacin (31.7%) with the lowest against Amikacin (13.6%). For *Escherichia coli*, the highest resistance rates were found to be 36.4% against Amikacin, 27.1% against Norfloxacin, and 26.8% against Ciprofloxacin. *Pseudomonas aeruginosa* demonstrated 31.8%, 24.4% and 21.3% resistance rates against Amikacin, Levofloxacin, and Nitrofurantoin, respectively (Table 5).

**TABLE 5:** Proportion (%) of uropathogens demonstrating Resistance (R) to antimicrobial agents.

Name of isolate	AU	CIP	CEF	GEN	PIP	NIT	NAL	CFT	NOR	TET	AMK	LEV
**Gram‐negatives**												
*Citrobacter spp*	15 (8.6)	12 (9.4)	12 (7.9)	10 (12.2)	14 (8.4)	7 (7.9)	13 (7.6)	15 (8.8)	13 (8.4)	12 (8.1)	1 (4.5)	1 (2.4)
*Enterobacter spp*	6 (3.4)	4 (3.1)	3 (2.0)	2 (2.4)	4 (2.4)	2 (2.2)	4 (2.4)	4 (2.3)	4 (2.6)	2 (1.3)	0 (0.0)	2 (4.9)
*E. coli*	44 (25.3)	34 (26.8)	36 (23.8)	17 (20.7)	40 (24.0)	19 (21.3)	44 (25.9)	44 (25.7)	42 (27.1)	38 (25.5)	8 (36.4)	10 (24.4)
*Klebsiella spp*	52 (29.9)	38 (29.9)	50 (33.1)	27 (32.9)	54 (32.3)	28 (31.5)	51 (30.0)	54 (31.6)	43 (27.7)	45 (30.2)	3 (13.6)	13 (31.7)
*P. aeruginosa*	35 (20.1)	25 (19.7)	29 (19.2)	14 (17.1)	31 (18.6)	19 (21.3)	35 (20.6)	30 (17.5)	30 (19.4)	29 (19.5)	7 (31.8)	10 (24.4)
*Proteus spp*	13 (7.5)	7 (5.5)	11 (7.3)	7 (8.5)	13 (7.8)	11 (12.4)	11 (6.5)	12 (7.0)	11 (7.1)	13 (8.7)	3 (13.6)	4 (9.8)
**Gram‐positives**												
*Enterococcus spp*	0 (0.0)	1 (0.8)	1 (0.7)	1 (1.2)	1 (0.6)	0 (0.0)	1 (0.6)	1 (0.6)	1 (0.6)	1 (0.7)	0 (0.0)	0 (0.0)
*S. saprophyticus*	4 (2.3)	2 (1.6)	4 (2.6)	1 (1.2)	4 (2.4)	1 (1.1)	4 (2.4)	5 (2.9)	4 (2.6)	4 (2.7)	0 (0.0)	0 (0.0)
*S. aureus*	3 (1.7)	2 (1.6)	3 (2.0)	2 (2.4)	4 (2.4)	2 (2.2)	4 (2.4)	4 (2.3)	4 (2.6)	3 (2.0)	0 (0.0)	1 (2.4)
*S. epidermidis*	1 (0.6)	0 (0.0)	1 (0.7)	0 (0.0)	1 (0.6)	0 (0.0)	1 (0.6)	1 (0.6)	1 (0.6)	1 (0.7)	0 (0.0)	0 (0.0)
*Streptococcus spp*	1 (0.6)	2 (1.6)	1 (0.7)	1 (1.2)	1 (0.6)	0 (0.0)	2 (1.2)	1 (0.6)	2 (1.3)	1 (0.7)	0 (0.0)	0 (0.0)
**Total**	174 (34.5)	127 (25.2)	151 (30.0)	82 (16.3)	167 (33.1)	89 (17.7)	170 (33.7)	171 (33.9)	155 (30.8)	149 (29.6)	22 (4.4)	41 (8.1)

Abbreviations: AMK, amikacin; AU, augmentin; CFT, ceftazidime; CIP, ciprofloxacin; CEF, ceftriaxone; GEN, gentamicin; LEV, levofloxacin; NIT, nitrofurantoin; NAL, nalidixic acid; NOR, norfloxacin; PIP, piperacillin; TET, tetracycline.

## 4 Discussion

Emerging global reports indicate a shifting landscape of uropathogens in relation to antibiotic resistance. To facilitate effective treatment, it is strongly advised to conduct regular surveillance to gather comprehensive data on the trends of antibiotic susceptibility and resistance among these pathogens. Primary health care facilities serve as the first line of care for a significant proportion of the Ghanaian population, particularly people living in rural communities and deprived districts in the country. UTIs are among the leading causes of outpatient department attendance at primary health care facilities in Ghana. Unfortunately, the majority of these facilities are not adequately equipped to perform urine cultures and antimicrobial susceptibility testing. This often leads to indiscriminate prescription of antibiotics, which is one of the major drivers of the surge in AMR. The present study aimed to evaluate the prevalence as well as antibiotic sensitivity and resistance patterns of microorganisms isolated from urine samples of patients at Methodist Hospital, Wenchi. The prevalence of UTIs during the study period was 45.2%. This finding is higher than rates reported by earlier studies [22, 23, 24]. Although the prevalence of UTIs was considerably high in this study, it was lower than reports of other previous studies [25, 26]. Variations in demographics, socioeconomic factors, environmental conditions, cultural practices, and antibiotic resistance patterns may account for these disparities.

The present investigation demonstrated that enterobacteria constituted the predominant category of isolated pathogens, with *Klebsiella spp* and *Escherichia coli* emerging as the most commonly identified isolates. *Staphylococcus spp* were the most commonly identified gram‐positive organisms. These findings align with various studies conducted in Ghana [12, 27, 28, 29, 30]. The intestinal microbiota in humans is primarily composed of enterobacteria, which are acknowledged as a frequent contributor to autoinfection in UTIs.

With the exception of *Enterococcus spp* and *Staphylococcus epidermidis*, a greater number of bacterial isolates in the present study were identified in urine samples from female participants. The significant microbial presence observed in urine samples from females (74.6%), in contrast to 25.4% in males, aligns with previous studies that reported a higher prevalence of uropathogens in females [31, 32, 33]. The relatively high prevalence of UTIs observed among females within this study may be attributed to factors such as the anatomical differences between male and female urethras, close proximity of the female urethra to the anal region and inadequate personal hygiene practices.

The age distribution analysis revealed that individuals aged 20–40 years exhibited the highest infection rates (39.0%), in contrast to other age groups. This finding diverges from findings in previous investigations, which indicated high infection rates in individuals 60 years or older [25]. The recorded high prevalence in this age group could be attributed to increased sexual activity. During sexual intercourse, friction and movement can push bacteria living in the anogenital area towards the urethra, thus facilitating the entry of these microorganisms into the urinary tract.

Knowledge regarding the susceptibility patterns of etiological agents responsible for UTIs serves a dual purpose: it aids clinicians in choosing suitable pharmacological interventions for treatment and provides robust guidelines for the empirical management of infections. This is particularly crucial, as prompt treatment and intervention can avert potential complications. Amikacin was relatively active against all uropathogens in this present study. This finding is comparable to other studies that reported high efficacy of this antibiotic [27, 34]. Amikacin has been identified as the most potent antibiotic against a wide range of bacterial species, encompassing both gram‐positive and gram‐negative strains [35]. In contrast to other antibiotics such as Augmentin, Amikacin has been on the Ghanaian pharmaceutical market for a relatively short period and this may have contributed to the relatively low levels of resistance observed with this antibiotic. However, the nephrotoxicity associated with Amikacin makes it unsuitable, particularly in patients with renal impairment [36] and only prescribed when necessary. This partly accounts for the comparatively greater vulnerability of pathogens to this antibiotic in relation to other commonly utilized agents.

The emergence of resistance to antimicrobial agents has been noted since their early use, and it has increasingly become a worldwide issue [37]. Significant resistance to the recommended antibiotics for the treatment of UTIs was identified in this study. Every bacterial isolate demonstrated resistance to at least two antibiotics, supporting findings from a previous study [35]. In the present study, both gram‐negative and gram‐positive isolates demonstrated resistance to the antibiotics tested. *Klebsiella spp* showed the highest resistance rates to most antibiotics, with resistance to Ceftriaxone(33.1%), Piperacillin(32.3%), and Nitrofurantoin (31.5%). This indicates that *Klebsiella* spp are a critical contributor to the overall resistance burden. *Escherichia coli* demonstrated significant resistance to Ciprofloxacin (26.8%), Norfloxacin (27.1%), and Tetracycline (25.5%). These findings align with the prevalence of *Escherichia coli* resistance to fluoroquinolones globally. *Pseudomonas aeruginosa* exhibited moderate resistance across most agents, with resistance rates ranging from 17.1% (Gentamicin) to 20.6% (Nalidixic acid). These rates underscore the limited effectiveness of commonly used antibiotics against this pathogen. *Proteus spp* had generally lower resistance rates compared to *Klebsiella spp* and *Escherichia coli*, except for Nitrofurantoin (12.4%), where it exhibited relatively higher resistance. On the contrary, gram‐positive isolates showed markedly lower resistance rates compared to gram‐negative isolates. *Enterococcus spp* demonstrated minimal resistance across all antibiotics, with no resistance observed to Amikacin and Levofloxacin. *Staphylococcus saprophyticus* and *Staphylococcus aureus* exhibited slightly higher resistance to beta‐lactams (Ceftriaxone and Augmentin), but resistance to Amikacin and Levofloxacin was absent. *Streptococcus spp* had negligible resistance rates, with the highest resistance rate of 1.6% against Ciprofloxacin and Nalidixic acid. Overall, Amikacin demonstrated the lowest resistance rate (4.4%) across all isolates, making it a strong candidate for empirical therapy. The relatively high rate of AMR reported in this study is worrying. In Ghana, people can easily purchase antibiotics from most pharmacies and drug stores without valid prescriptions. This age‐old practice has culminated in the abuse and overuse of common antibiotics, leaving in its wake unacceptably high levels of AMR. It is incumbent upon the regulatory agencies to reclassify some of these antibiotics from over‐the‐counter to prescription‐only status to stem this tide.

Multi‐drug resistance (MDR) in this study was defined as resistance of the isolates to three or more antimicrobial agents [38]. At least 8 (72.7%) of the 11 bacterial isolates demonstrated MDR. *Klebsiella spp* demonstrated MDR to beta‐lactams (Ceftriaxone ‐ 33.1%, Ceftazidime ‐ 31.6%), fluoroquinolones (Ciprofloxacin ‐ 29.9%, Norfloxacin ‐ 27.7%), and tetracyclines (Tetracycline ‐ 30.2%). Additionally, resistance to nitrofurans (Nitrofurantoin ‐ 31.5%) and aminoglycosides (Gentamicin ‐ 32.9%) was observed, further emphasizing its ability to resist multiple antimicrobial agents and thus making it difficult to treat infections caused by this uropathogen. *Escherichia coli* demonstrated MDR to beta‐lactams (Ceftriaxone ‐ 23.8%), fluoroquinolones (Ciprofloxacin ‐ 26.8%, Norfloxacin ‐ 27.1%), and tetracyclines (Tetracycline ‐ 25.5%). Notably, resistance to the nitrofuran class (Nitrofurantoin 21.3%) and aminoglycosides (Gentamicin ‐ 20.7%) was also significant. *Pseudomonas aeruginosa* demonstrated resistance to Ceftriaxone (19.2%), Ciprofloxacin (19.7%), Gentamicin (17.1%), and Nitrofurantoin (21.3%), thereby meeting the criteria for MDR. *Proteus spp, Citrobacter spp*, and *Enterobacter spp* demonstrated some level of MDR. None of the gram‐positive isolates showed evidence of MDR. Again, none of the isolates demonstrated extensive drug resistance, as all retained susceptibility to at least two antimicrobial agents. Concerted effort is required to address the high rate of MDR recorded in this study. Clinicians are advised to prescribe antibiotics only when they are indicated and for the appropriate duration. Again, there is a need to intensify public education about the dangers associated with the indiscriminate use of antibiotics, as well as the importance of completing the full course of antimicrobial agents whenever they are prescribed. Also, the Food and Drugs Authority, together with other relevant agencies, should consider issuing administrative orders to restrict the sale of over‐the‐counter antibiotics to only persons in possession of valid prescriptions.

To mitigate the recurrence of UTIs in female patients, several proactive measures can be recommended. Boosting immunity with Vitamin D may reduce infection risk, while cranberry juice helps prevent bacterial adhesion in the urinary tract. For menopausal women, estrogen cream application in the vaginal area supports microbiota restoration, lowering UTI recurrence [39, 40, 41].

In this study, *Candida spp* were isolated from 5.8% of urine samples with 89.7% from urine samples of female patients. Several studies have reported the isolation of *Candida spp* from urine samples [42, 43, 44]. Their presence in urine culture poses a diagnostic challenge, requiring differentiation between colonization, contamination, and true infection. *Candida spp* are opportunistic fungi, often residing as commensals in the genitourinary tract, especially in individuals with risk factors like prolonged catheterization, diabetes mellitus, antibiotic use, or immunosuppression. The clinical relevance of candiduria remains context‐dependent. While asymptomatic cases may not require treatment, symptomatic patients need further evaluation for potential fungal UTIs or systemic infection. This study highlights the role of antimicrobial stewardship in preventing opportunistic infections, as broad‐spectrum antibiotic overuse promotes fungal overgrowth. Additionally, nosocomial transmission remains a concern, reinforcing the need for strict infection control measures such as hand hygiene and catheter care in healthcare settings.

This study is the first of its kind to be conducted on antimicrobial susceptibility patterns of uropathogens at the Methodist Hospital, Wenchi and also one of the few studies conducted in a primary healthcare facility in Ghana. Its findings are expected to aid clinicians in selecting effective antimicrobial agents, monitoring resistance trends, and guiding empirical therapy. Despite these advantages, the study also presents some limitations. The study relied on archived data, which may have limited the completeness and accuracy of the information collected. Additionally, the study was conducted at a single primary care hospital, potentially limiting the generalizability of the findings to other healthcare settings or populations in Ghana. Moreover, only a limited range of antibiotics was tested, potentially excluding other effective treatment options. The absence of quantitative fungal colony counts further constrained the ability to distinguish between colonization and true infections in cases involving *Candida spp*.

## 5 Conclusion

The study underscores the high prevalence of UTIs at Methodist Hospital, Wenchi. Gram‐negative bacteria were the predominant uropathogens, with *Klebsiella spp* and *Escherichia coli* demonstrating significant resistance to commonly used antibiotics. Amikacin emerged as the most effective antibiotic, demonstrating the lowest resistance rates. However, the alarming levels of MDR among isolates highlight the need for evidence‐based antimicrobial policies, routine surveillance, and strengthened infection prevention and control measures. These findings contribute valuable data to guide empirical treatment and promote antimicrobial stewardship in similar healthcare settings.

## Author Contributions

**Samuel Kyeremeh Adjei:** conceptualization, methodology, data curation, writing – original draft, writing – review and editing, formal analysis. **Prosper Adjei:** writing – review and editing.

## Ethics Statement

Ethical approval was obtained from the Research Ethics Committee of Methodist Hospital Wenchi before commencement of the study (Ethics approval reference: MHW/RE/103). The study did not incorporate any identifying details such as patient name or identification number, ensuring that all data was handled with the highest level of confidentiality. Additionally, the requirement for informed consent was waived by the Research Committee.

## Conflicts of Interest

The authors declare no conflicts of interest.

## Transparency Statement

The lead author, Samuel Kyeremeh Adjei, affirms that this manuscript is an honest, accurate, and transparent account of the study being reported; that no important aspects of the study have been omitted; and that any discrepancies from the study as planned (and, if relevant, registered) have been explained.

## Data Availability

The data that support the findings of this study are available from the corresponding author upon reasonable request.
